# The Influence of Telemedicine Care on the Management of Behavioral and Psychological Symptoms in Dementia (BPSD) Risk Factors Induced or Exacerbated During the COVID-19 Pandemic

**DOI:** 10.3389/fpsyt.2020.577629

**Published:** 2020-09-15

**Authors:** Walter Barbalho Soares, Ingrid Tinôco Silvestre, Amannda Melo de Oliveira Lima, Katie Moraes de Almondes

**Affiliations:** ^1^ Psychosocial Care Unit, Onofre Lopes University Hospital, Federal University of Rio Grande do Norte, Natal, Brazil; ^2^ Private Practitioner, Natal, Brazil

**Keywords:** elderly, behavioral and psychological symptoms in dementia, social isolation, caregiver burden, COVID-19, telemedicine, dementia, BPSD

## Abstract

The number of people with dementia worldwide is expected to increase to approximately 1.3 billion in 2050. Almost 90% of patients diagnosed with dementia suffer from behavioral and psychological symptoms of dementia (BPSD). BPSD causes and risk factors are multiple and complex and can be responsible for hospitalizations in long-term institutions, psychiatric hospitalizations and search for health services. Recently, the world imposition of social distance and self-isolation as the best preventive measures for the COVID-19 pandemic has created challenges in the health care and management of this population, which may trigger or aggravate BPSD, and most caregivers are not prepared to address it. In face of this actual social distancing, telemedicine comes to be a tool for improving the management of these acute symptoms and mental care. In this article, we discuss and disseminate recommendations on this important alternative of assistance, especially considering the cases of BPSD. In this context of a pandemic, even patients with BPSD and caregivers require more frequent and updated guidance, considering the difficult context to social distance. Telemedicine can reduce the risk for the development of negative outcomes in mental health precipitated by the reduction of social contact and less access to health services, improving dementia symptom management, mainly BPSD.

## Introduction

Dementia, according to the Diagnostic and Statistical Manual of Mental Disorders 5th edition, is a neurocognitive disorder defined as a chronic and gradually developing decline of cognitive functions that results in occupational and social dysfunctions (1). The number of people with dementia worldwide is expected to increase to approximately 1.3 billion in 2050 (2, 3). Even if cognitive symptoms are commonly considered the hallmark feature of dementia, patients usually present a wide variety of “non-cognitive” neuropsychiatric symptoms, and they are important disease manifestations (4, 5). They are termed behavioral and psychological symptoms of dementia (BPSD) and represent a heterogeneous group of non-cognitive symptoms related to disturbed perception, thought content, mood, and behavior.

Throughout the course of the dementia process, the vast majority of patients will develop one or more of these symptoms, which can include agitation, motor disturbance, anxiety, irritability, depression, apathy, disinhibition, hallucinations, delusions, and sleep or appetite disturbances (6–9), and their prevalence may increase from mild to severe dementia (10). Almost 90% of patients suffer from dementia (11), although the etiology and management of dementia are still a challenge, and dementia can be responsible for increased referrals to nursing homes and prolonged periods of hospitalization (12).

A preliminary study rated BPSD in 124 patients with Alzheimer’s disease and found that the prevalence of neuropsychiatric symptoms in this population was higher for apathy (51%), dysphoria (50%), and irritability (38%) (11), while another study with 408 patients evaluating a 5-year period prevalence of neuropsychiatric symptoms in dementia found that this was greatest for depression (77%), apathy (71%), and anxiety (62%) (13). The progression of the severity of dementia increases the likelihood of hallucinations while decreasing the odds of depression, and Alzheimer’s disease (AD) patients, compared to other dementias, are less likely to present agitation, disinhibition, and depression (14).

Coronavirus disease 2019 (COVID-19) is an infectious respiratory illness caused by SARS-CoV-2, a newly emergent coronavirus that was first identified in Wuhan, China at the end of 2019 (15) and declared a pandemic in March 2020 (16). Recently, the world imposition of social distance and self-isolation as the best preventive measures for COVID-19 has created challenges in health care and management, especially for elderly people (17). In this context, telemedicine, a remote medical practice using telecommunication and information technologies, appeared to be a viable alternative to face-to-face consultations (18).

In this article, we aim to discuss the worsening of BPSD in elderly people with dementia during the pandemic and defend how telemedicine can be an important alternative for the current context.

## ELDERLY PEOPLE and THEIR HIGH RISK for SOCIAL ISOLATION

After the outbreak of COVID-19, the increase in the amount of information about the disease and concerns about its implications are impacting global mental health (19). The increase in the number of suspected cases and confirmed patients spread the public worry of being infected. The uncertain future of the pandemic has been exacerbated by the excess of information, sometimes driven by erroneous news reports (20). Sick patients may experience fear of an uncertain prognosis due to the fatal potential of the virus. On the other hand, the general population, especially those who are experiencing quarantine, can feel boredom, loneliness, and anger (21). This situation can be stressful for all people, provoking fear and anxiety about the disease and causing strong emotions in children and adults (22).

In fact, these changes and the fear of the unknown lead to increased psychiatry symptoms in both healthy people and those with pre-existing mental health problems (19). The stress associated with COVID-19 increases the chances of patients with pre-existing mental disorders to relapse or have new episodes. Therefore, it is important to find a balance between distance and social isolation since the loneliness imposed by quarantine can cause harmful psychological effects, especially for the elderly (23).

Elderly people can experience these feelings more intensely. They already have special physical, psychosocial, and environmental vulnerabilities associated with age (24). Case fatality in individuals 65 years or above is higher than that in other populations (25). Their frailty brings the risk of various infections and decreases the immune response; they have more comorbidities and more hospitalizations, increasing the chance of being infected with COVID-19 (26). Knowledge about this vulnerability can increase the effect of the uncertainty and fear of the pandemic, and they may experience the fear of their own death and of losing their loved ones (24).

The known limitations of the elderly in dealing with technology gadgets and sensory and cognitive deficits may make it difficult for them to access updated information about COVID-19 situations, making them victims of misinformation and inadequate precautionary measures to follow (24). Furthermore, self-isolation as a preventive measure can severely affect the elderly whose only social contact is out of home; those who do not have the support of their families or friends and depend on the social support of volunteers or social care could be in additional risk situations, along with those who live alone or isolated (27). Social distancing can be an independent risk factor for depression, anxiety and suicide, especially in places such as nursing care or old-age homes (28). Studies have observed that under these stress factors, the level of anxiety among nursing homes and caregivers increased, and they developed signs of exhaustion and burnout after a month of full lockdown (17).

Beyond age, patients with dementia are more susceptible to morbidity and mortality of the virus because they have more cardiovascular disease, diabetes, and pneumonia compared to the elderly without dementia (29). Other features may increase the risk of contracting COVID-19, such as the difficulties of this population to follow the recommendations from public health to prevent the transmission: correct hand hygiene, maintain physical distance, monitor and report symptoms of the disease and self-isolation by remaining alone at home (15).

In addition to social isolation, elderly people infected with COVID-19 could have experienced other consequences, including hospitalization and behavioral problems. One of the main symptoms of the disease is dyspnea, and the hypoxia generated by COVID-19 can cause delirium, which may complicate the course of dementia, increasing the suffering of the patients and their caregivers, the cost of medical care, and the need for support (17).

Increased demand in the health care system can hinder the access of patients with chronic diseases, such as dementia, to the services, and the fear of being infected during the use of health care settings can impair outpatient follow-up and the use of emergency services if necessary. The workup, diagnosis and clinical follow-up of these patients can be harmed by deviation of resources and professionals to act in combat of pandemic, and those living alone in community may feel loneliness due the social isolation and absence of their group activities (28).

## The Pandemic Can Increase Risk Factors for Worsening Behavioral Symptoms

BPSD causes and risk factors are multiple and complex. Factors that contribute to the occurrence of these symptoms can be categorized as follows: factors related to the patient (neurobiological changes—brain lesions and type of dementia, changes in neurotransmission and neuromodulation, acute medical illness—urinary tract infection, pneumonia, dehydration, constipation, unmet needs—pain, sleep problems, fear, pre-existing personality and psychiatric illness); caregiver factors (stress, burden, depression, lack of education about the disease, communication issues) and environmental factors (safety issues, overstimulation or under stimulation, lack of structure or lack of established routines) (8, 9).

BPSD is not well diagnosed, and the treatment is poorly understood. Deciding which aspects constitute a behavioral disorder is extremely subjective and is associated with worse cognition, more severe stages of dementia, high levels of distress both in patients and their caregivers (family members or health professionals), long-term hospitalization, misuse of medications and increased health care costs (6).

Neuropsychiatric manifestations could be divided into three different groups according to the main symptoms presented: affective syndrome, psychological syndrome, and other neuropsychiatric disorders (30). Some studies reveal that there are differences between the occurrences of BPSD over time. In general, hyperactivity and apathy have high persistence and incidence, depression and anxiety have moderate incidence, low or moderate persistence, and psychotic symptoms are less prevalent with a moderate or low incidence (10). This is important for the identification and proper approach by the doctor and caregivers.

The initial management of BPSD is to identify and quantify the symptoms to evaluate the possibility of being secondary to comorbidities such as infection, dehydration, metabolic decompensation, adverse drug effects, and others. Proper treatment of these comorbidities alone can mitigate BPSD. If those are not the causes, non-pharmacological measures have to be instituted. Under normal conditions, environmental adaptations or modifications, the establishment of a specific routine, guidance for caregivers and family members and programs for physical activity, music and light therapy are good strategies for dealing with these symptoms (30). Some of these can be harmed during the pandemic, which can become a problem for non-pharmacologic management of these conditions.

In the actual context, several risk factors (social isolation; pharmacology adherence; caregivers’ burden; reduction of nonpharmacologic strategies; lack of medical evaluation; modification of house routine) can arise to trigger or exacerbate BPSD. Elderly people, especially those who are isolated and with cognitive decline or dementia, can become more anxious, irritated, stressed, agitated, and withdrawn during quarantine. Most of the caregivers are not prepared to deal with BPSD, requiring guidance on where and how to get practical help and regular medications.

## Telemedicine as a Viable Alternative Tool for Elderly Mental Healthcare

Telemedicine is defined as a tool to provide healthcare at a distance through the use of telecommunications technology (31). The first reference to telemedicine was in 1897, informing the use of telephone calls instead of a personal doctor visit for a bedridden home patient (32). Today, many people have telecommunication devices, such as smartphones, laptops and tablets that could be used as private real-time consultations (33).

Moreover, telemedicine is growing in popularity because of the COVID-19 pandemic context and social distance (34). After all, in addition to social isolation, there are still restrictions on public transport, which also represents a major barrier to access medical care (35). It is an alternative tool that could be more used and enable mental health professionals to keep improving health care during the outbreak (36, 37). Additionally, the elderly are affected by health problems that need frequent monitoring, and telemedicine, by breaking geographical barriers and reducing unnecessary travel, facilitates access and management of all these factors by the caregivers, improving health care (38).

It is important to consider that most elderly people need a caregiver (professionals, family, friends) who must be supported by health services (23) and benefit from telemedicine. Caregivers have many responsibilities in caring for dementia patients, and the convenience and accessibility of telemedicine could help them manage psychosocial issues and even their own support, doubts and early interventions (31).

Therefore, telemedicine is well established in the literature as an alternative balance between social distance and the need for specialized consultation. It can imply cost reduction, relief of the health system, less exposure of the population at risk to infections, continuity of monitoring during the period of social distance and greater articulation between health services and patients/caregivers (31, 37, 39).

This system allows for easy access to a dementia specialist and can assist the patient in maintaining his clinical stability, as well as providing caregivers with sufficient guidance to deal with new symptoms that may appear during the pandemic, relieving stress caused by BPSD. Additionally, it expands access to clinical resources and links health care providers with patients and their caregivers, thereby overcoming the limitations of face-to-face appointments. In addition, telemedicine may reduce hospitalization and emergency department visits (40) and implies higher rates of treatment continuation in dementia patients, which could suggest that telemedicine improves factors that can contribute to slowing the progression of dementia, resulting in better prognosis, reduced hospitalization and visits to urgency/emergency settings (37).

Although psychiatry evaluation is about what we see, nonverbal communication and what is not said (36), mental health is still a specialty that can be well suited to telemedicine programs (31). Studies show that telemedicine has a high level of satisfaction and effectiveness with low cost, is very convenient, and is easily accessible (41). In addition, it provides clinical outcomes equivalent to face-to-face services (31, 37, 39). Telemedicine is not just a replacement for face-to-face appointments; it holds the possibility of new avenues for care delivery, more frequent but shorter encounters, and opportunities for earlier intervention (41) to improve mental health.

## Telemedicine Challenges

Studies have listed some problems with telemedicine, such as technically challenged staff (11%), resistance to change (8%), cost (8%), age of patient (5%), and level of education of patient (5%) (31). Additionally, it is important to include visual and hearing problems of elderly people as a difficult factor in handling electronic devices, and we should provide appropriate adjustments to them (33).

There are many platforms to use, but for Brazil’s public health reality, due to the low education and social level of our population (especially in our reality—University Hospital), WhatsApp is probably the most accessible mobile app. Even so, we still face some other problems related to infrastructure and population needs, such as: some of them have no smartphone to proceed a video call, poor internet connection, and the need of a caregiver to help with the telecommunication process.

In those cases, we need to lay hands on a simple phone call, but we have been successful considering the pandemics’ needs. This tool works well for established patients, or the one we already know, but for the first time, it could be insufficient (36). For a complex or difficult case, we still proceed with a face-to-face consultation, respecting the rules of individual protection.

## Discussion

The COVID-19 pandemic has brought important structural and behavioral changes worldwide. For health systems, social distancing has imposed the need for new alternatives for medical care without exposing patients, especially risk groups such as the geriatric population, to possible infections. The elderly have more reason to suffer from this whole situation, both in relation to their biological risk of contracting the disease and to the more restricted social isolation. Those with dementia are at serious risk of losing their effective follow-up, adjusting medications and general orientations when non-pharmacological approaches should be adopted. In addition, the lack of routine and of the others outside therapeutic alternatives can worsen dementia symptoms, especially BPSD.

There are a few reasons why BPSD can worsen during a pandemic, such as social isolation, caregiver stress, sleep disturbances, lack of medical follow-up with medication adjustments, changes in the house routine, risk of infections, and untreated clinical diseases. One of the most important points in the treatment of BPSD is non-nonpharmacological interventions, which involve social and physical contact, such as social and exercise groups. The social isolation imposed by the pandemic suspended these interventions and will also result in decreased social engagement and worse disease progression. It is necessary to create new alternative plans for these patients in this new situation (**Table 1**).

**Table 1:** Recommendations for old age people with cognitive impairment and their caregivers in times of COVID-19 pandemic (42, 43).

1. Public health information can be difficult to understand—Try to transmit the information in a clear and simple way, remembering how to properly do the hygiene measures as many times as necessary and helping the elderly to do it. Using memory aids like pictures or notes can assist in this task; encourage and celebrate the small achievements.
2. Keep in touch with family and friends through electronic devices so that the elderly do not feel so lonely.
3. Look for signs of impaired mental health (is he feeling more anxious? Sad? Confused)? and provide psychological support, encouraging them to talk about any feelings and look for professional help if it is necessary.
4. Changes in routine can be difficult and increase BPSD: try to maintain a routine as similar as before the pandemic, the activities you would usually do around the house and keep to regular meal and bedtimes.
5. Learn simple physical exercises to do at home with the elderly to maintain the mobility. Relaxation and mindfulness are good activities too.
6. Try to promote cognitive stimulation (listen to music, see family photos and try to remember who are in those, discuss special objects); stimulate light activities, like taking care of plants and animals.
7. Take care of a good sleep routine.
8. Create a week schedule and do the plans to maintain the routine.
9. Be sure of the amount of medications and groceries you have at home so that you are safe.
10. Look for the possibilities of medical assistance by telemedicine. Ask all the questions you have; ask for help to manage the symptoms at home and make sure there is enough medication at home.
11. Have easy access to all possible help channels: close family, taxis, phone number, supermarket, medical assistance.

In this context, telemedicine comes as a valid alternative to expand access to clinical resources and links health care providers with patients and their caregivers, thereby overcoming the limitations of face-to-face appointments and being a balance between social distance and the need for specialized consultation.

In contrast to the results found from the latest Kaiser Family Foundation (KFF) Health Tracking Poll (a survey project that provides consistent and up-to-date information on the public’s opinions, knowledge and experiences with the U.S. healthcare system), that seven out of 10 adults 65 and older (68%) say they have a computer, smartphone or tablet with Internet access at home, but only 11% of this population says they used one of these devices for a teleconsultation; in our services, we have achieved greater adherence to psychogeriatric telemedicine during this pandemic period. During the month of May 2020, we performed 34 teleconsultations through video (WhatsApp) or audio (phone call); 45 were scheduled, only five did not answer the contact, one patient died, two rescheduled because of first appearance in our service, two had wrong schedules, and one was not confirmed. In June and July, we made 43 and 58 consultations, respectively, and the vast majority of them were by teleconsultation. In the same period of last year (May, June and July, 2019), our psychogeriatric service had 51, 42, and 25 scheduled consultations, respectively, 42, 33, and 24 of which were made.

The service flow of our service begins three or four days before the consultation day. Our nurse team has contact with the patient or a caregiver, telling him that he will receive a psychiatric teleconsultation and communicate our orientations for a good interaction (for example, the patient must be with a caregiver at home; they should be in a quiet room; test the connection). Initially, we had a poor adherence level, and this nurse contact was very important to increase it and decrease the time lost to explain these orientations during medical contact. Most of the patients or caregivers were open to this type of medical care and tried to make it work. Important prescription modifications have been made during these pandemic months, such as new depressive episodes, psychotic episodes, or the worsening of BPSD, which probably avoided more severe symptoms. When we have doubts about medical conduct, the patient was scheduled to receive an ambulatory consultation. Nevertheless, some of the caregivers had no compromise with the teleconsultation; by the time of scheduled communication, they were not with the patient. The application of scales for cognitive screening was another important difficulty, which became a limitation. Patients and caregivers had trouble understanding our instructions to download, answer, or perform some scale tasks.

A recent survey study used a television-based telehealth service to support European adults living with mild dementia or mild cognitive impairment and found that those who are using this technology are doing more memory exercises and can help them with cognitive stimulation in this particular situation. Access to these devices may reduce feelings of isolation, and the creation of a specific telephone support line has been described as effective in providing health information and social support to this population (44).

Telemedicine might help reduce the risk for the development of negative mental health outcomes precipitated by a reduction in social contact and less access to health services, improving dementia symptom management, mainly BPSD, and mental care. In addition, it can help caregivers by providing more agile guidance on non-pharmacological measures to control symptoms adapted to the new reality of confinement. Additionally, it allows health support in real time, even at a distance, making possible adequate medication adjustment, when necessary, without exposing the patient and caregiver to risks of infection.

## Data Availability Statement

The original contributions presented in the study are included in the article/supplementary material; further inquiries can be directed to the corresponding authors. 

## Author Contributions

Author contribution study design: KA, WS. Writing the draft: KMA, WS, IS, AL. Integration of the authors’ comments: KA, WS, IS, AL. Final manuscript: KA, WS, IS, AL. All authors contributed to the article and approved the submitted version.

## Conflict of Interest

The authors declare that the research was conducted in the absence of any commercial or financial relationships that could be construed as a potential conflict of interest.
